# Side bias behaviour in dogs (*Canis familiaris*) is influenced by task complexity

**DOI:** 10.1038/s41598-025-11192-7

**Published:** 2025-07-23

**Authors:** Anna Kis, Hannah Roper, Henrietta Bolló, Antónia Balogh, Anna Gergely, József Topál

**Affiliations:** 1 Psychobiology Research Group, HUN-REN Research Centre for Natural Sciences, Institute of Cognitive Neuroscience and Psychology, Budapest, Hungary; 2 ELTE-HUNREN NAP Comparative Ethology Research Group, Budapest, Hungary; 3 Department of Psychology and Physical Education, Apor Vilmos Catholic College, Budapest, Hungary; 4 Doctoral School of Biology, ELTE Eötvös Loránd University, Budapest, Hungary; 5 Department of Ethology, ELTE Eötvös Loránd University, Budapest, Hungary

**Keywords:** Animal model, Choice task, Side bias, Task complexity, Coevolution, Social evolution

## Abstract

Side bias is often reported in canine cognition experiments when dogs’ behaviour is tested in two-way choice tasks. Recently it has been proposed that side bias, which remains consistent over time and across different situations, may parallel a human psychiatric condition known as visual neglect. Here we tested an additional factor that could contribute to the development of side bias behaviour in dogs: task complexity. Subjects were tested in a series of two-way choice tasks of varying difficulty and the proportion of subjects showing side bias was compared across these tasks. Results showed that dogs were more likely to develop side bias in more complex situations, such as the food preference task, or in more unusual tasks, such as elbow pointing, compared to simpler tasks like sustained pointing. This suggests that, as expected, task complexity contributes to dogs’ side bias, with side preference potentially being at least partly triggered by more challenging tasks that yield decreased reward.

## Introduction

Dogs are among the most studied species in animal cognition research due to their special evolutionary history, during which they adapted to the human social environment^1,2^. Pioneering studies that first suggested human-analogue socio-cognitive skills in dogs, predominantly used simple two-way object choice tasks to demonstrate that dogs, like human infants, are capable of responding to human communicative signals, e.g. following human pointing gestures^3^. This together with the finding that dogs’ social communication skills are more human-like than our closest primate relatives^4^, has led to considerable interest in dog cognition. Comparative research continues to use two-way choice tasks still today e.g.^5,6^.

Findings about dogs’ special susceptibility to human communication have been consistently replicated over the years (e.g. pointing^7,8^, although see^9^). However, a less emphasised phenomenon in dog cognition literature is that a considerable proportion of subjects are excluded from the analysis prior to reporting these findings. One of the main reasons for ignoring a subject’s performance (other than procedural errors or the subjects’ unwillingness to participate) is side-preference. Dogs that consistently choose one side over the other are often excluded from the analysis. However, the criteria for what constitutes side-preference vary between studies ranging from excluding dogs with a 100% left or right choice (e.g.^10^) to excluding those that consistently choose one side significantly more often than the other (e.g.^11–13^). As a result, varying percentages of subjects are removed before data analysis (80% in^12^, 7% in^10^). The rationale behind excluding these subjects is understandable: dogs with side-preference base their choices on something other than the cues (e.g. pointing gestures) provided in the experiment. These subjects, however, are still motivated to participate in the experiment and to obtain the offered reward (such as food or a toy), thus their performance remains a response to the experimental situation, even if not the response the experimenters intended to investigate.

The question of why side-preference, which is often considered undesirable from the researchers’ perspective, emerges so often in dogs has not yet been properly addressed. This is despite the fact side-preference is prevalent in a wide range of species. For example, Damaraland mole-rats *(Fukomys damarensis)* showed a left-turning side bias in a T-maze task, both at the individual and population levels^14^. In domestic chicks *(Gallus gallus)* it was observed that asymmetric hatching behaviour had a significant effect on later side bias in turning and footedness^15^. Honeybees *(Apis mellifera)* showed a rightward side bias when entering an open cavity, which greatly affected colony-level decision-making^16^. Cape sugarbirds *(Promerops cafer)* and the lesser double-collared sunbird *(Nectarinia chalybea)* also displayed individual side biases in feeder choice tasks regardless of environmental changes, which was considered stereotyped foraging behaviour^17^. In ants *(Lasius niger),* a consistent left-turning side bias was observed in a Y-maze task independent of context^18^. Recent research^19^ suggests, that at least in case of dog subjects showing a strong and consistent side-preference, this may be an attentional bias, similar to the visual neglect in humans^20,21^. We have to note, however, that lateralised spatial attention in itself is not a pathological condition^22^. There is also a considerable amount of research suggesting that lateralisation, similar to handedness in humans, occurs in dogs at the individual level, although there is no consistent population-level bias^23,24^. Lateralisation has been argued to serve adaptive functions^25^, and a similar reasoning for side-preference in two-way choice tasks suggests that consistently choosing one side could yield a considerable amount (50%) of reward. While earning a 100% reward would be better, if the task is overly complex, always choosing one side might be a more profitable strategy instead of high cognitive investment which would still lead to lower reward. In primates, there is evidence that task complexity influences handedness^26,27^. Here we investigate how the proportion of dogs showing side-preference change as a function of task complexity or difficulty.

## Methods

### Ethics statement

Research was approved by the National Animal Experimentation Ethics Committee (Ref No. XIV-I-001/531-4-2012) and was done in accordance with the Hungarian regulations on animal experimentation and the guidelines for the use of animals in research described by the Association for the Study Animal Behaviour (ASAB). All owners volunteered their dog to participate in the study and they gave written informed consent. The sample size (N = 23) was determined based on the sample sizes used in previously published studies employing similar test procedures (N = 16 in^28^ and N = 29 in^19^).

### Subjects

N = 23 privately owned pet dogs (11 males, 6 neutered & 12 females, 10 neutered; mean ± SD age: 6.41 ± 3.03 years) from various breeds and mongrels were recruited on a voluntary basis.

### Test procedure

All subjects completed a total of six different two-way choice tasks, which varied in difficulty based on data from the literature. Each task comprised of six trials, yielding a total of 36 choices per subject.

A spontaneous choice between two baited containers was used as a baseline measure of the subjects’ preference without any experimenter-given cue^19^. This was followed by five additional tasks in a random order (Table 1). An array of pointing-following tasks^28^ were administered including an easier sustained pointing task (where the experimenter’s hand remains pointed at the baited location until the dog makes its choice), a more difficult momentary pointing task (where the experimenter withdraws her pointing hand before the dog is released), and an even harder (ambiguous) elbow pointing task (where the experimenter’s elbow points to the baited container, but her hand/finger points to the non-baited container). Pointing following tasks typically use a semi-random baiting for the left and the right sides, so that the first and the second correct choices are on different sides, and no side is baited more than twice consecutively. Such procedures are intended to prevent subjects from developing a side bias. In the present experiment, we deliberately included not only the widely used semi-random baiting order (right-left-left–right-right-left or left–right-right-left-left–right for both momentary and elbow pointing), but also a blocked order (right-right-right-left-left-left or left-left-left–right-right-right for sustained and momentary pointing) to examine the potential effect of trial order arrangement. Additionally, a more complex food preference (quantity discrimination) task^29^ was also included, where subjects had to choose between two plates containing one versus eight food pellets (visible to the dogs). The specific choices for the conditions administered were based on literature data. The blocked procedure was added in the first place, as it is routinely reported in publications that trial randomisation is applied to avoid the development of side bias, but actually there is no published data on the blocked version. The momentary pointing condition was chosen to be administered with both random and blocked trial order as dogs are reported to perform above chance in this task (with random trial order), but below ceiling, thus there is room for both increased and decreased success due to the applied manipulation (blocked trial order). Sustained pointing is reported to be the easiest for dogs to complete, thus we expected that dogs would perform at ceiling in the randomised version of this task (which is used in literature), and we only included the blocked version—mainly to have another task with blocked trial order to match the sustained pointing. Elbow pointing and numerical discrimination is reported to be hard for dogs to follow, even with the random trial order.

**Table 1:** The sequence of the different tasks for each subject.

ID	Spontaneous	Sustained RRRLLL	Momentary RLLRRL	Momentary RRRLLL	Elbow point RLLRRL	Food Pref. RLLRRL
1	1	2	4	3	5	6
2	1	6	3	2	4	5
3	1	5	2	6	3	4
4	1	4	6	5	2	3
5	1	3	5	4	6	2
6	1	2	3	5	6	4
7	1	4	5	2	3	6
8	1	6	2	4	5	3
9	1	5	6	3	4	2
10	1	3	4	6	2	5
11	1	6	4	5	3	2
12	1	3	6	2	5	4
13	1	2	5	6	4	3
14	1	4	2	3	6	5
15	1	5	3	4	2	6
16	1	6	5	3	2	4
17	1	4	3	6	5	2
18	1	2	6	4	3	5
19	1	3	2	5	4	6
20	1	5	4	2	6	3
21	1	2	3	4	5	6
22	1	3	4	2	6	5
23	1	4	6	5	3	2

Spontaneous choice was the first task for all subjects, while the remaining tasks were randomly distributed from 2nd to 6th.

### Data analysis

For each subject, the number of left and right choices made (ranging from 0 to 6) was recorded for each of the six tasks, which is presented as descriptive data. A side preference score was then calculated with dogs making 3 left and 3 right choices receiving a score of 0 (3–3 = 0), while dogs making 6 left and 0 right or 0 left and 6 right choices receiving a score of 6 (6–0 = 6). This way each dog had a side preference score ranging from 0 to 6 for each of the six tasks, reflecting how biased their choices were toward one side, without distinguishing whether the preferred side was the left or the right.

Subjects’ side preference scores were then compared across conditions with paired samples nonparametric tests (Wilcoxon sign rank tests) using SPSS version 22.

## Results

**Fig. 1:**
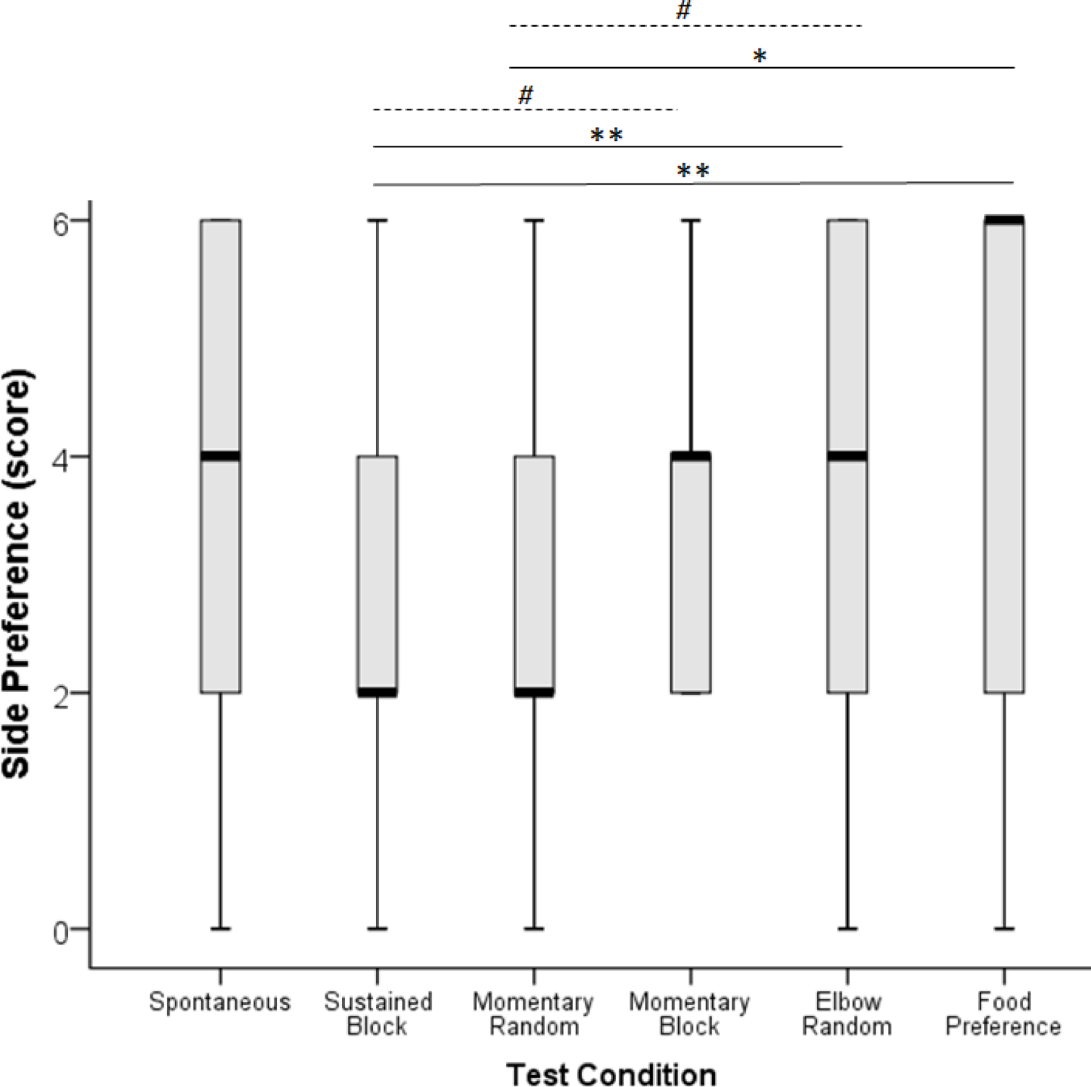
Dogs’ side preference score across the six tasks (median, quartiles and whiskers). **: *p* < 0.01, *: *p* < 0.05, #: *p* < 0.1.

Side preference scores for each of the test conditions are shown in Fig. 1. It was found that dogs in the *Food Preference* task were most prone to side bias (median side preference score = 6), and their scores significantly differed from those in the *Sustained Pointing with blocked trial order* (Z = 2.881, *p* = 0.004) and *Momentary Pointing with random trial order* (Z = 2.089, *p* = 0.037). However no significant differences were observed between *Food Preference task* and *Elbow Pointing* or *Momentary pointing with blocked trial order* (both *p* > 0.1). Side preference scores for *Elbow Pointing* (median = 4) were similarly higher than those for *Sustained Pointing with blocked trial order* (Z = 2.594, *p* = 0.009) and tended to be higher than those for *Momentary Pointing with random trial order,* though this difference was not significant (Z = 1.750, *p* = 0.080). *Elbow Pointing*, however, did not differ significantly from *Momentary pointing with blocked trial order* (Z = 0.728, *p* = 0.476). The difference between *Sustained Pointing with blocked trial order* (median = 2) and *Momentary pointing with blocked trial order* (median = 4) did not reach statistical significance (Z = 1.904, *p* = 0.057) and no difference was found between *Sustained Pointing with blocked trial order* and *Momentary Pointing with random trial order* (Z = 0.847, *p* = 0.382). Finally, subjects’ side preference score in the *Spontaneous choice* task did not differ from any of the other five tasks (all *p* > 0.1).

Looking at the descriptive data for individuals who showed a 100% side bias (6/6 choices to the same side) in any of the tasks, we found that there was N = 1 dog that exhibited side bias in the spontaneous task, but not in any of the cued tasks (false positive). The remaining dogs that showed side bias in the spontaneous task also showed side bias in at least one of the cued tasks. On the other hand, among the N = 5 dogs that showed side bias in at least one of the cued tasks, but not in the spontaneous tasks (false negatives), only N = 1 individual showed side bias in an “easy” task (momentary blocked pointing), while all N = 5 of these dogs displayed side preference in the “hard” tasks (elbow pointing or food preference).

**Fig. 2:**
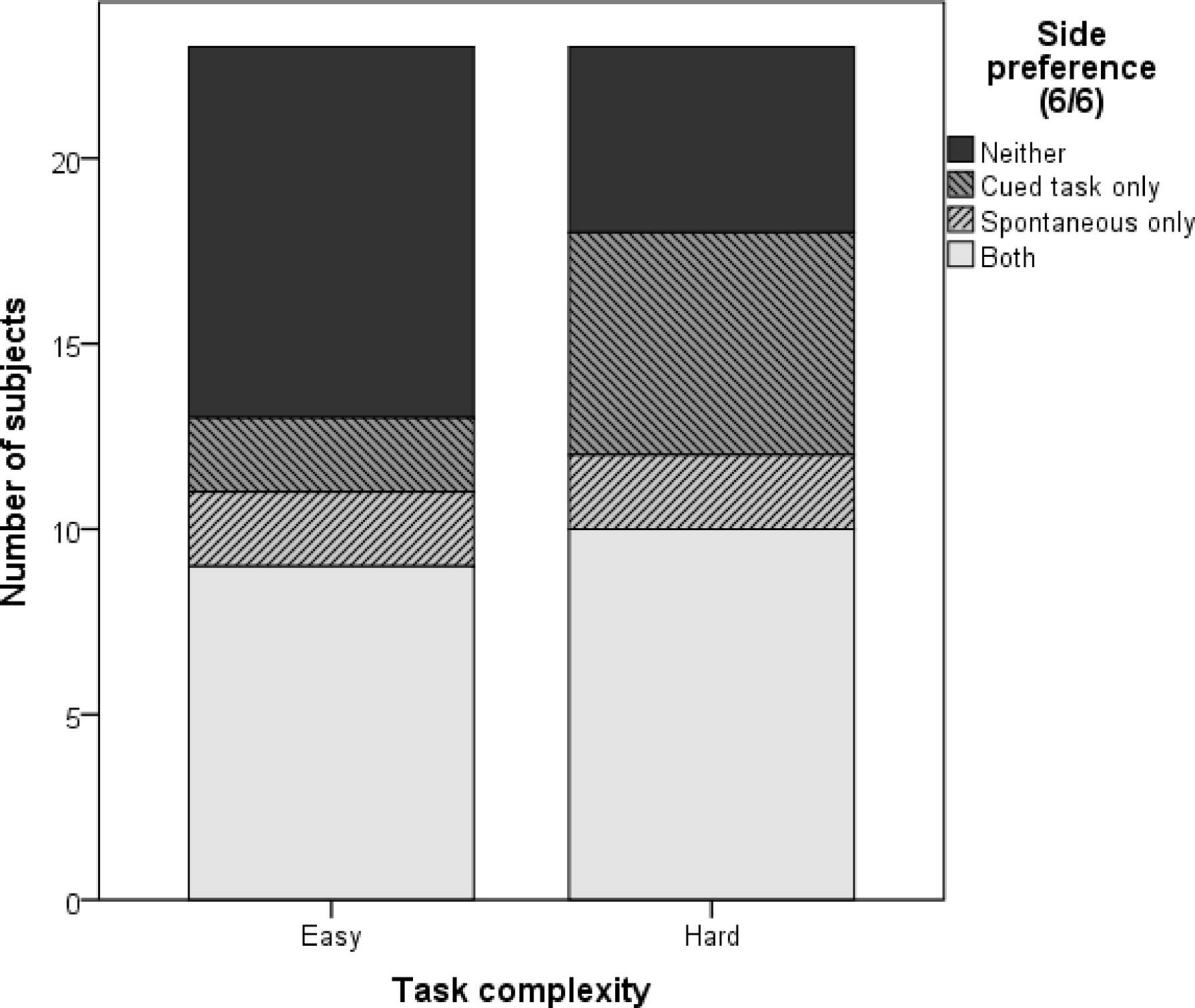
Number of dogs with a 100% side preference in (1) both the spontaneous and the cued task, either (2) the spontaneous or (3) the cued task only, and (4) neither of the tasks. Data are shown separately for the three easy cued tasks (sustained pointing, momentary blocked pointing, momentary random pointing) and the two hard cued tasks (elbow pointing, food preference). A dog is considered to show side preference in the easy or hard cued category if it displayed a 6/6 biased choice in any task within that category.

The distribution of subjects (Fig. 2) that showed a side preference (1) in both the spontaneous and at least one cued task, (2) the spontaneous task only, (3) the cued task only, or (4) neither of the two differed from random for the easy cued tasks (sustained pointing, momentary blocked pointing, momentary random pointing; χ^2^ = 9.870, *p* = 0.020), but not for the hard cued tasks (elbow pointing, food preference; χ^2^ = 5.696, *p* = 0.127).

**Fig. 3:**
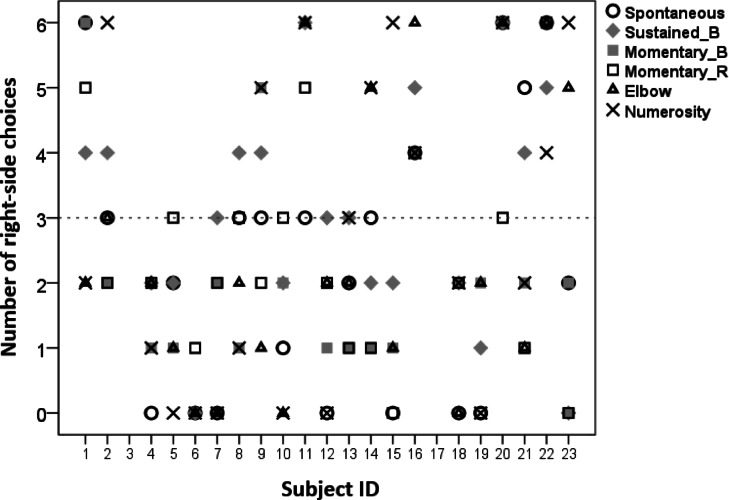
Number of right-side choices as a function of the different two-way choice tasks for each individual subject. Dashed line depicts 50% chance level. 6/6 and 0/6 scores are individually significant (binomial test, *p* < 0.05).

Regarding the (left or right) direction of the side preference no within-individual consistency was found across tasks (Fig. 3), which replicates previous results on dogs’ motor laterality^30^.

## Discussion

The results of the current study show that dogs’ side-preference differs between two-way choice tasks of varying difficulty. This is consistent with human studies showing that side-preference is more prevalent in more complex tasks^31^. Intuitively, this suggests that dogs are likely to follow the experimental cue when it is easy to obtain a high percentage of the reward but may switch to a side-preference strategy when the task becomes more difficult. This finding is important not only to understand the factors behind side-bias behaviour in dogs, but also because it suggests that by excluding subjects showing a side-preference from datasets, animal cognition researchers may inadvertently bias their results.

The percentage of subjects categorised as showing a side bias varies across published studies, partly due to differing definitions of side bias). In the current study, the percentage of subjects with a 100% side bias is similar to previous research (see e.g.^19^ for simple two-way choice tasks, and^32^ for a food preference task). Our results further show that dogs’ side bias scores in the spontaneous side preference test (used as a diagnostic tool in^19^ do not differ from either the easy or the hard tasks tested in the current study. The number of false positive results, however, is satisfactorily low (N = 1 out of 23 subjects), dogs that would be identified as “side-preferenced” based on the spontaneous test, do indeed show side preference in the cued tests. A further indirect difference was found between the easy and hard tasks in terms of subjects showing 100% side bias. According to our results, while dogs in the easy tasks may rely on the experimenter-given cues to counteract their spontaneous side preference, the opposite occurs in the hard tasks, where even subjects who do not show a spontaneous side preference often switched to choosing one side consistently. This finding resonates with the side preference scores, and further supports the role of task complexity inducing side bias.

Lateralised behaviour in dogs is generally variable and not necessarily consistent even within individuals^30^. Such inconsistency in the left/right direction of side preference was also replicated in the present study. This parallels human findings, where certain tasks elicit stronger hand preferences than others^33^. For example, people may have a strong preference for their dominant hand when writing, but a much weaker preference for which hand they use to turn on the light. The same argument has been supported in rodents, the prevalence of side bias in mice increases with lower visual discriminability^34^, and an abrupt drop has been observed in the amount of side-biased choices preceding the onset of successful discrimination^35^. Side or spatial attentional bias in dogs has been proposed to be a trait-like feature (e.g. co-varying with paw preference;^36^. However, the present results along with the widely replicated inconsistency in dogs’ paw preference suggest that side bias may be, at least in part, a strategic response employed by dogs in situations where e.g. the experimental cues are overly complex. This could lead to an arbitrary pattern of left versus right preferences. Of note, there have been documented cases of dogs showing a side bias consistent in time and across situations^19^, but for the typical population tested in the current study, this is not the case. An additional factor to consider is that a shift towards ambilaterality was detected in dogs experiencing acute stress, induced by an open field test environment compared to their home setting^37^. It is reasonable to assume that increased task complexity could elevate stress levels which might interfere with the emergence of side bias and exert an opposing effect to the side-bias inducing effect of task complexity.Dogs have been suggested to be excellent models for human behaviour, including several psychiatric disorders^38,39^. There are fundamental differences in dogs’ and humans’ organisation of visuospatial processing, e.g. there is no evidence in dogs for the right hemisphere dominance widely reported for humans^36^. Still, dogs’ side-preference behaviour in some aspects resembles human hemispatial neglect, making them a promising model for this condition^19^. The current results emphasize that task complexity should be considered when assessing side bias in dogs, and suggest that side bias as a choice strategy should be distinguished from an inability to attend to one side of the space.

## Data Availability

All research data related to this publication is stored in the HUN-REN server and is available upon request from the corresponding author.
